# Supplementary material for: Efficient electron transmission in covalent organic framework nanosheets for highly active electrocatalytic carbon dioxide reduction
Source: Nat Commun. 2020 Jan 24;11:497. doi: 10.1038/s41467-019-14237-4 (PMC6981265; doi:10.1038/s41467-019-14237-4)
Supplement: Supplementary file 1 — Supplementary Information [file 41467_2019_14237_MOESM1_ESM.pdf]

Supplementary Information for

Efficient electron transmission in covalent organic framework nanosheets for highly active electrocatalytic carbon dioxide reduction

Zhu et al.

Outline

1. Supplementary Figures (Figure. 1-42) .... 3-44.

2. Supplementary Tables (Table 1-2) .... 45-46.

3. Supplementary Notes .... 47-48.

4. Supplementary References .... 49.

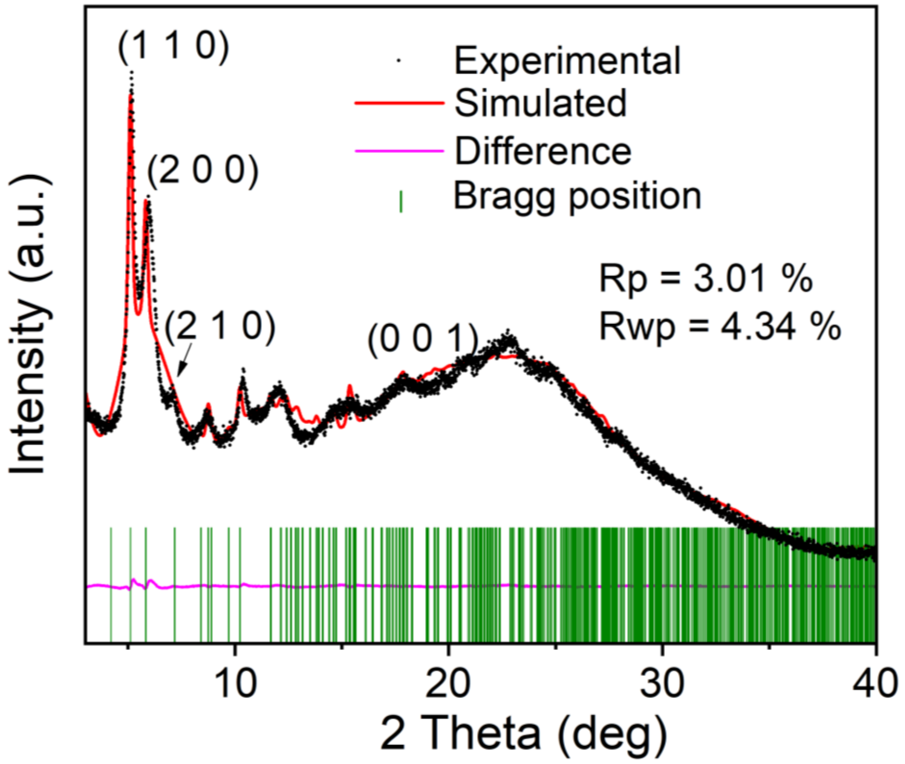

Supplementary Figure 1. Experimental (black dot) and simulated (red line) PXRD patterns of Co-TTCOF.

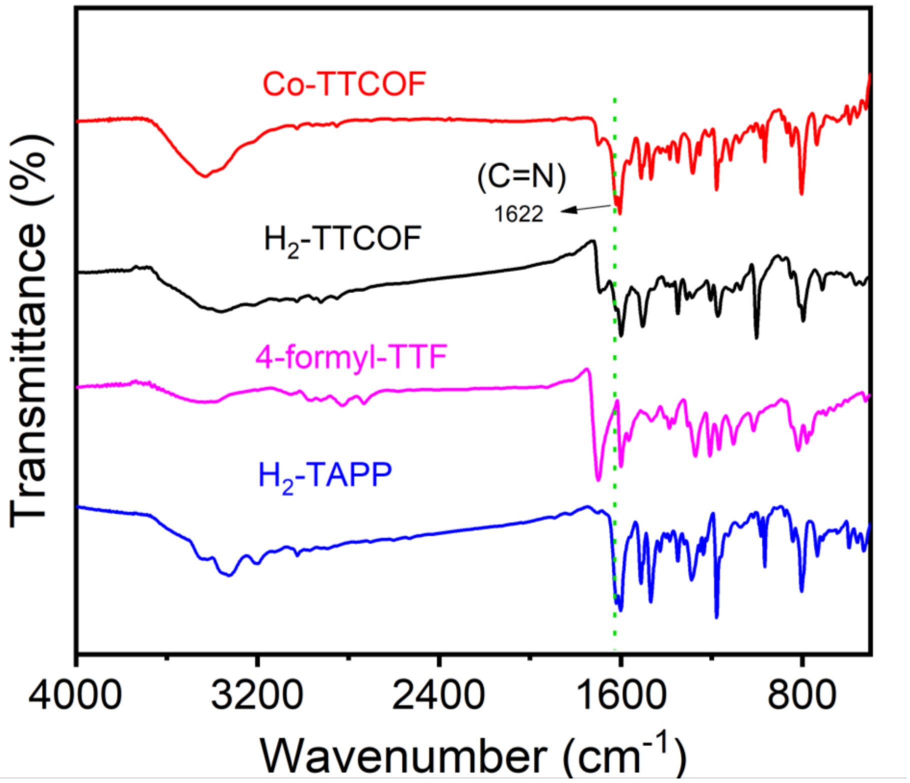

Supplementary Figure 2. FT-IR spectra of Co-TTCOF, H_2-TTCOF, 4-formyl-TTF and H_2-TAPP.

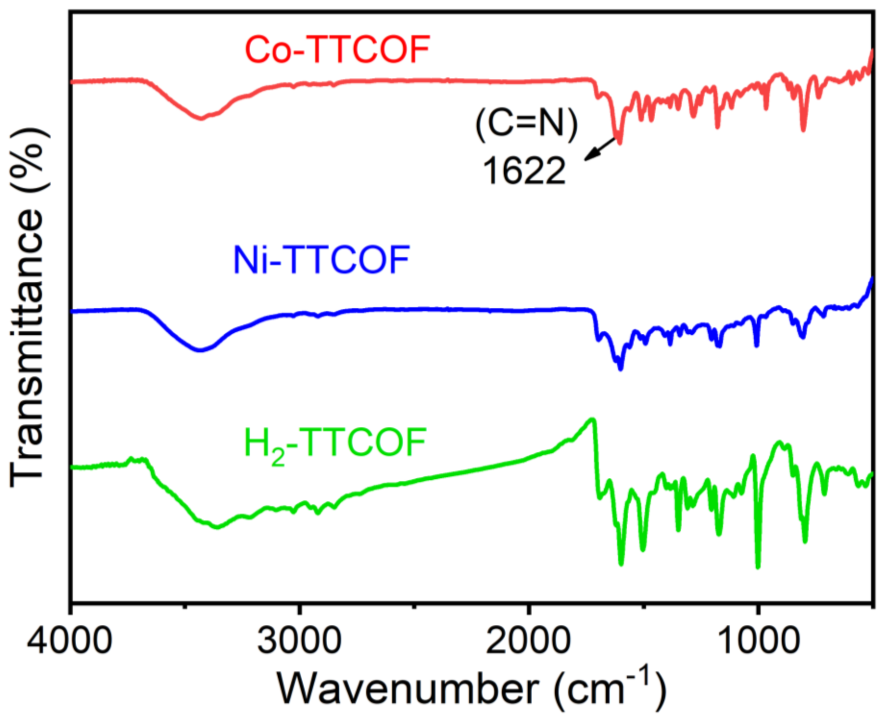

Supplementary Figure 3. FT-IR spectra of Co-TTCOF, Ni-TTCOF and H_2-TTCOF.

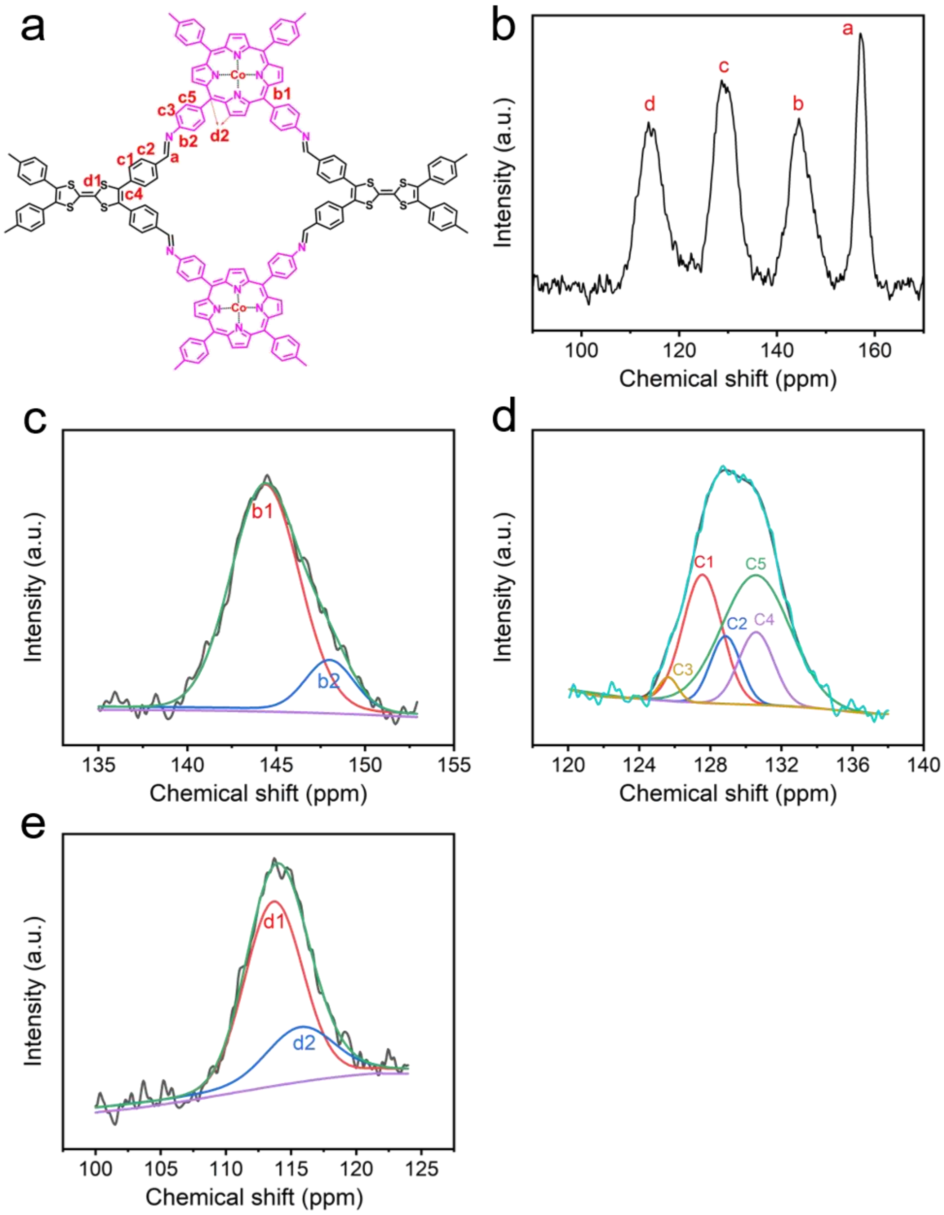

Supplementary Figure 4. Representative ^{13}C cross polarization solid-state NMR spectra of Co-TTCOF. **a** The schematic structure of Co-TTCOF. **b** Solid state ^{13}C NMR spectrum of Co-TTCOF. **c-e** The spectral deconvolution of Co-TTCOF in **b**. The test frequency was 600 MHz in a ^{13}C NMR (Bruker AVANCE III 600).

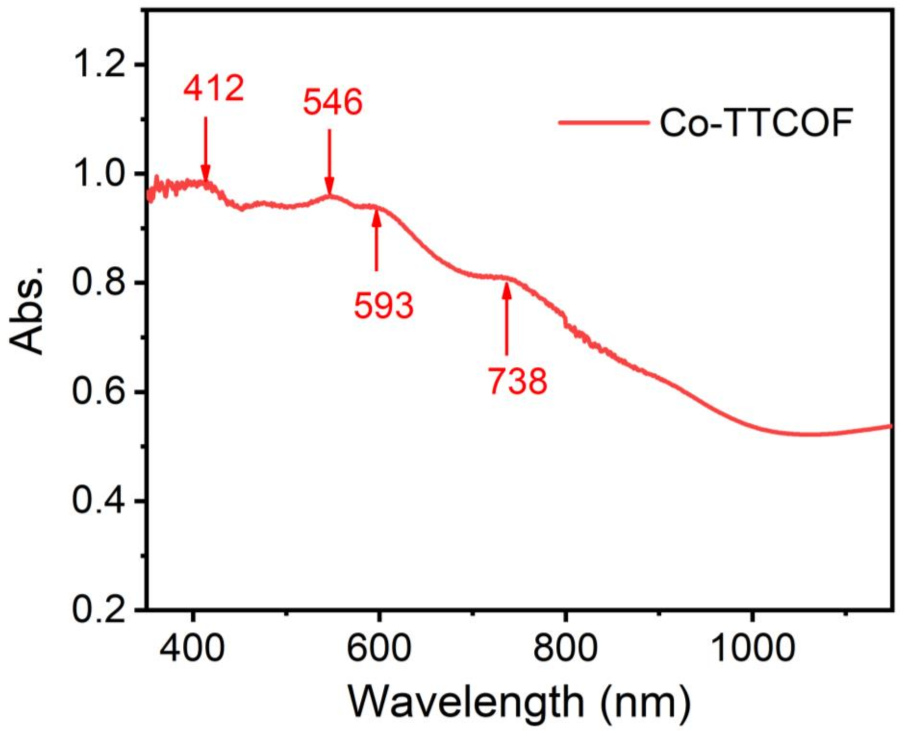

Supplementary Figure 5. The solid state UV spectra of Co-TTCOF.

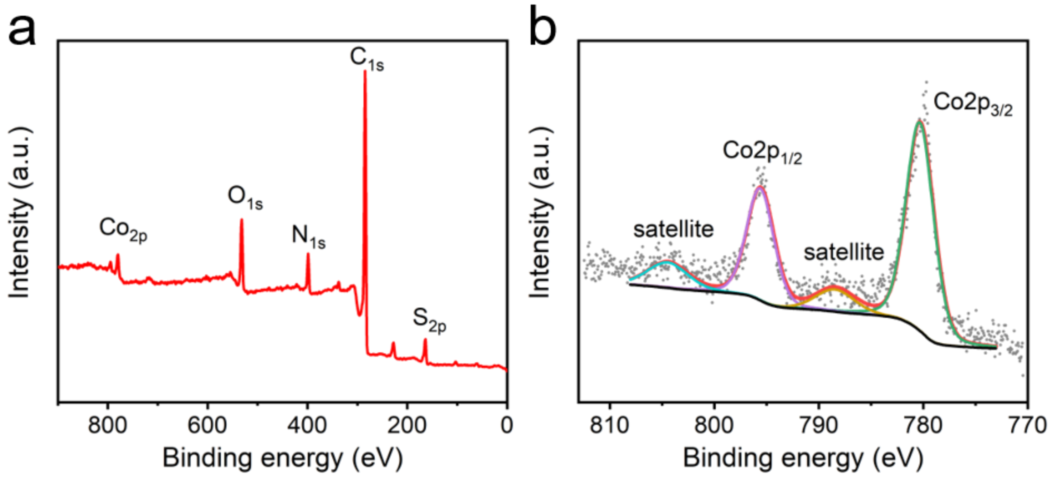

Supplementary Figure 6. High-resolution XPS spectrum of Co-TTCOF. **a** Total XPS spectra of Co-TTCOF. **b** XPS high-resolution scan of Co 2p.

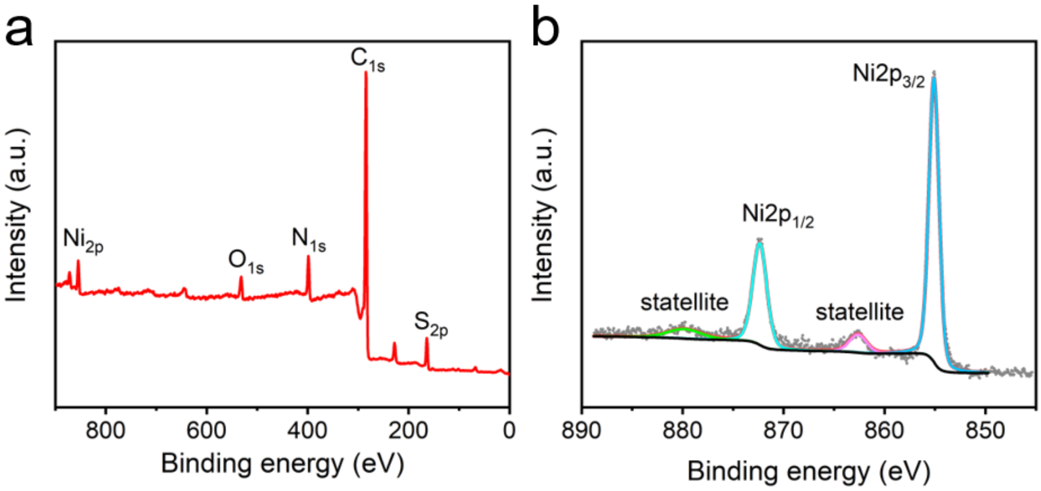

Supplementary Figure 7. High-resolution XPS spectrum of Ni-TTCOF. **a** Total XPS spectra of Ni-TTCOF. **b** XPS high-resolution scan of Ni 2p.

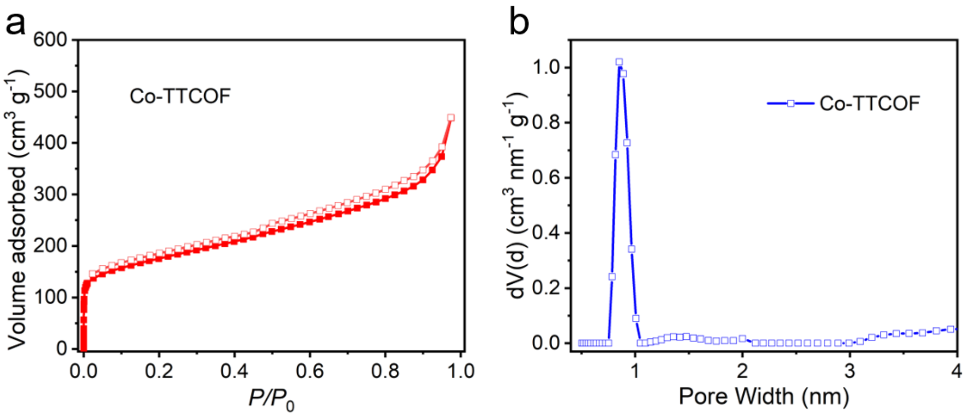

Supplementary Figure 8. N_2 sorption results of Co-TTCOF. **a** N_2 sorption isotherm curve of Co-TTCOF. BET surface area was calculated based on the P/P_0 range of 0.01-0.15 (r = 0.999991). **b** Pore size distribution of Co-TTCOF. The result is fitted to N_2 at 77 K on carbon (slit/cylindr. pores, QSDFT adsorption branch). The fitting error was 0.305 %.

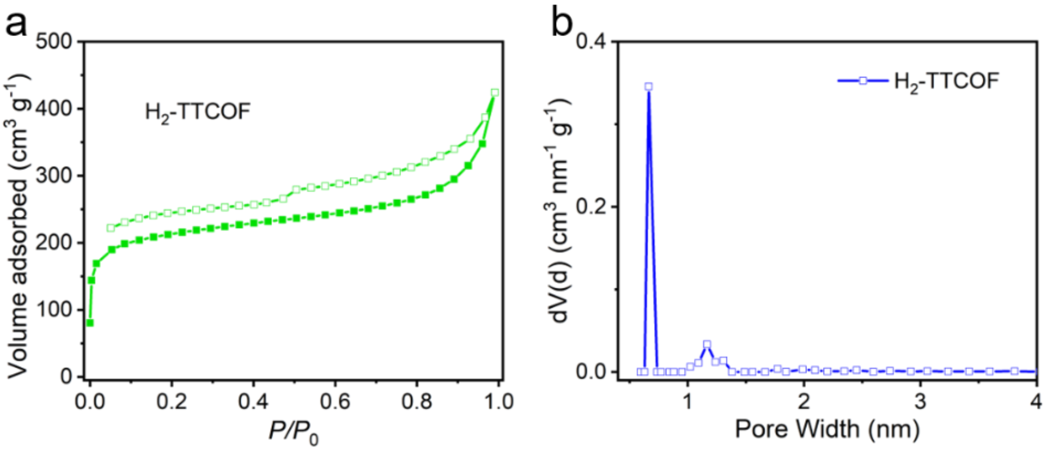

Supplementary Figure 9. N_2 sorption results of H_2-TTCOF. **a** N_2 sorption isotherm curve of H_2-TTCOF. BET surface area was calculated based on the P/P_0 range of 0.05-0.29 (r = 0.997829). **b** Pore size distribution of H_2-TTCOF. The result is fitted to N_2 at 77 K on carbon (slit/cylindr. pores, QSDFT adsorption branch). The fitting error was 0.564 %.

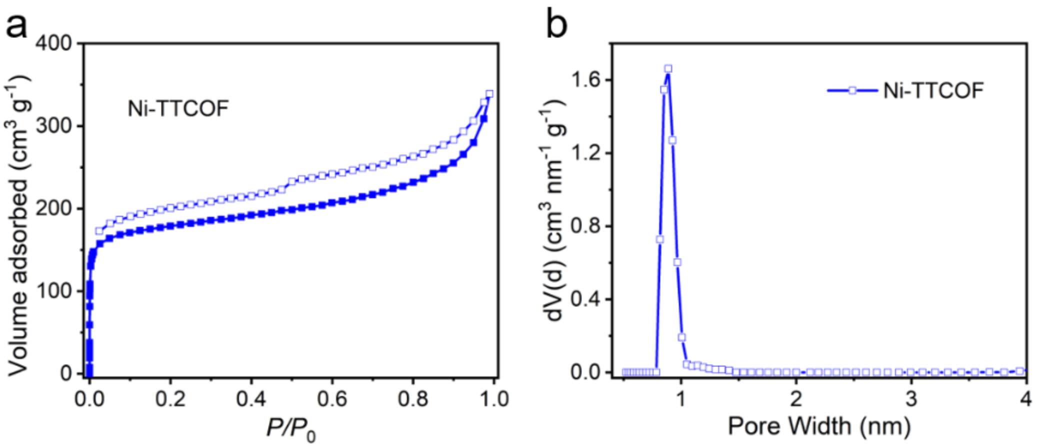

Supplementary Figure 10. N_2 sorption results of Ni-TTCOF. **a** N_2 sorption isotherm curve of Ni-TTCOF. BET surface area was calculated based on the P/P_0 range of 0.003-0.049 (r = 0.999995). **b** Pore size distribution of Ni-TTCOF. The result is fitted to N_2 at 77 K on carbon (slit/cylindr. pores, QSDFT adsorption branch). The fitting error was 0.475 %.

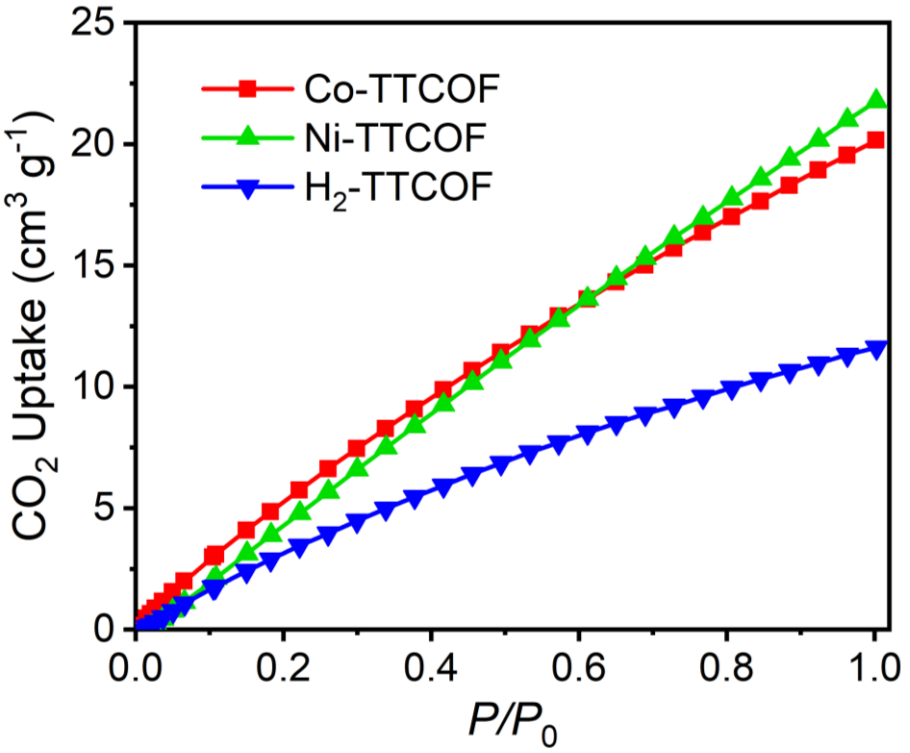

Supplementary Figure 11. CO_2 adsorption curves of M-TTCOFs at 293 K.

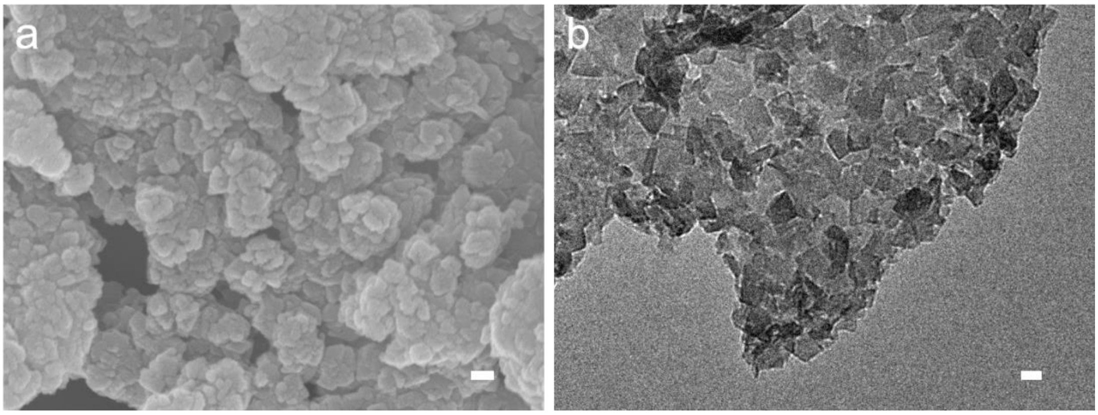

Supplementary Figure 12. SEM and TEM images of H_2-TTCOF, scale bar = 100 nm. **a** SEM image. **b** TEM image.

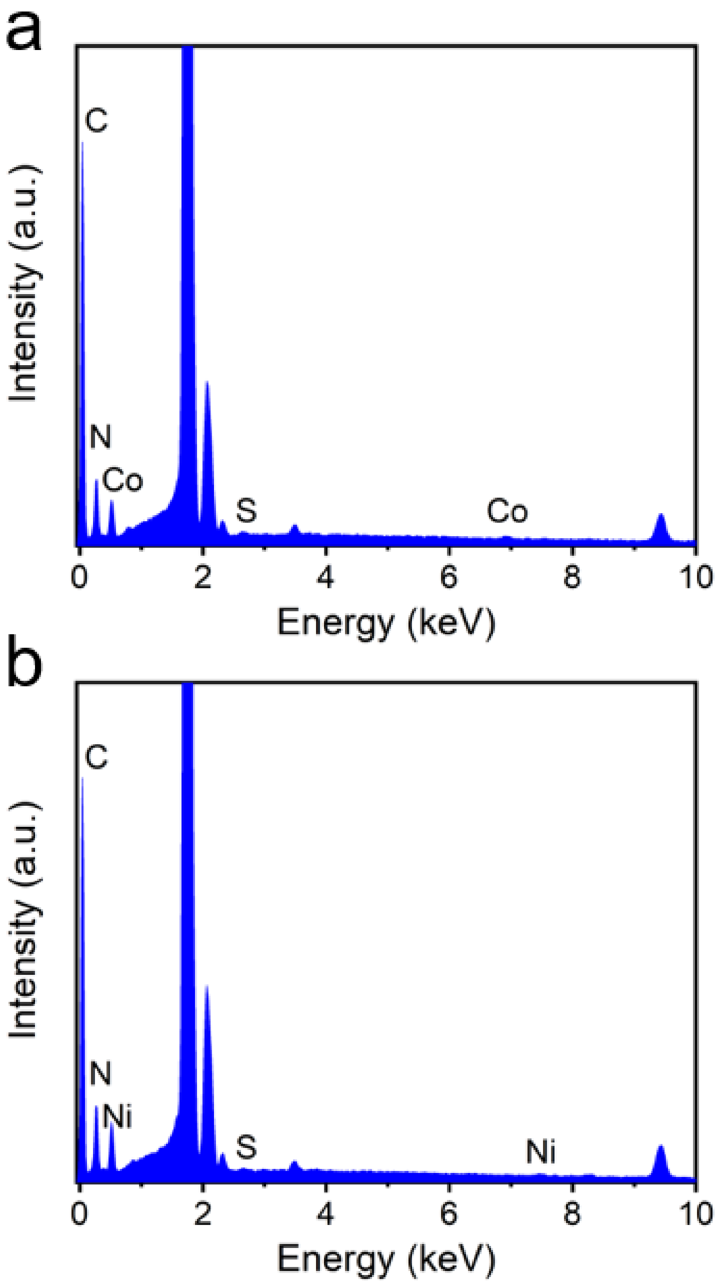

Supplementary Figure 13. EDS spectra of M-TTCOFs. **a** Co-TTCOF. **b** Ni-TTCOF.

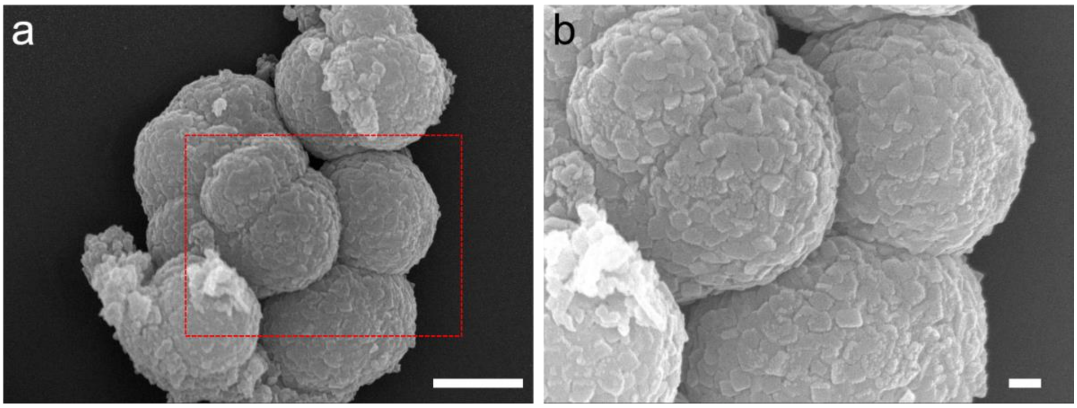

Supplementary Figure 14. SEM images of Ni-TTCOF, scale bar = 1 μm. **a** SEM image. **b** The enlarged place in **a**.

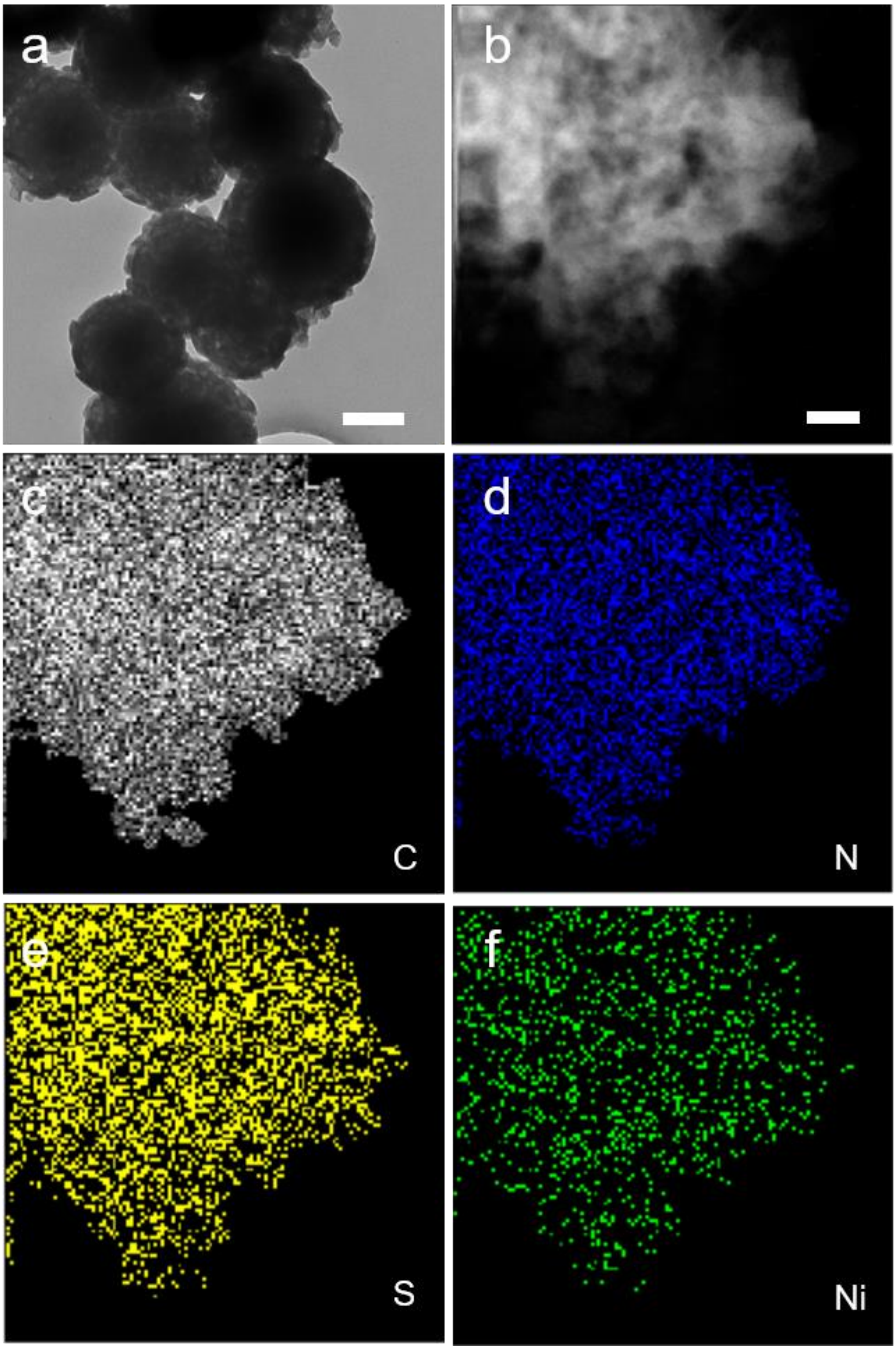

Supplementary Figure 15. Morphology characterization of Ni-TTCOF. **a** TEM image, scale bar = 250 nm. **b** STEM-HAADF image, scale bar = 50 nm. **c-f** Corresponding element mapping images for C, N, S and Ni.

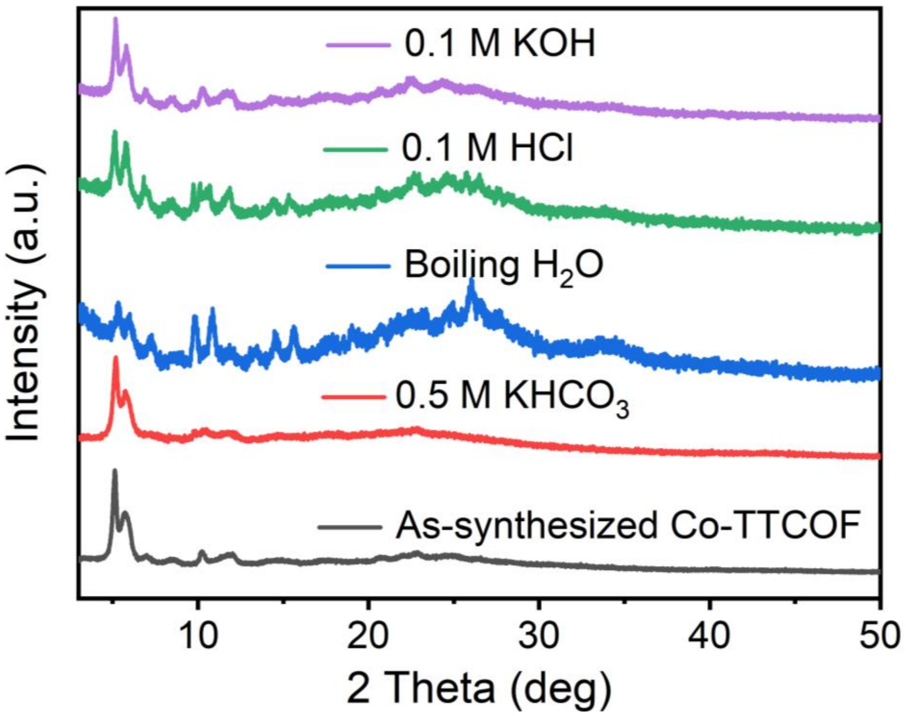

Supplementary Figure 16. PXRD patterns of Co-TTCOF after chemical stability tests. In the tests, Co-TTCOF was immersed in these solutions for more than 5 days.

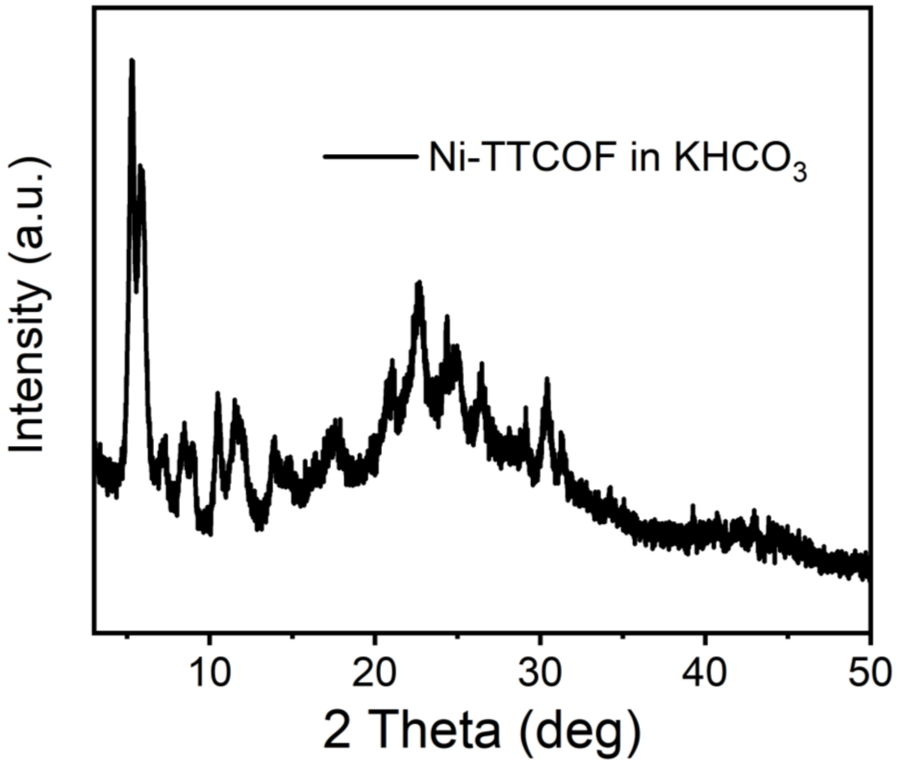

Supplementary Figure 17. PXRD pattern of Ni-TTCOF after immersing in 0.5 M $KHCO_3$ for 5 days.

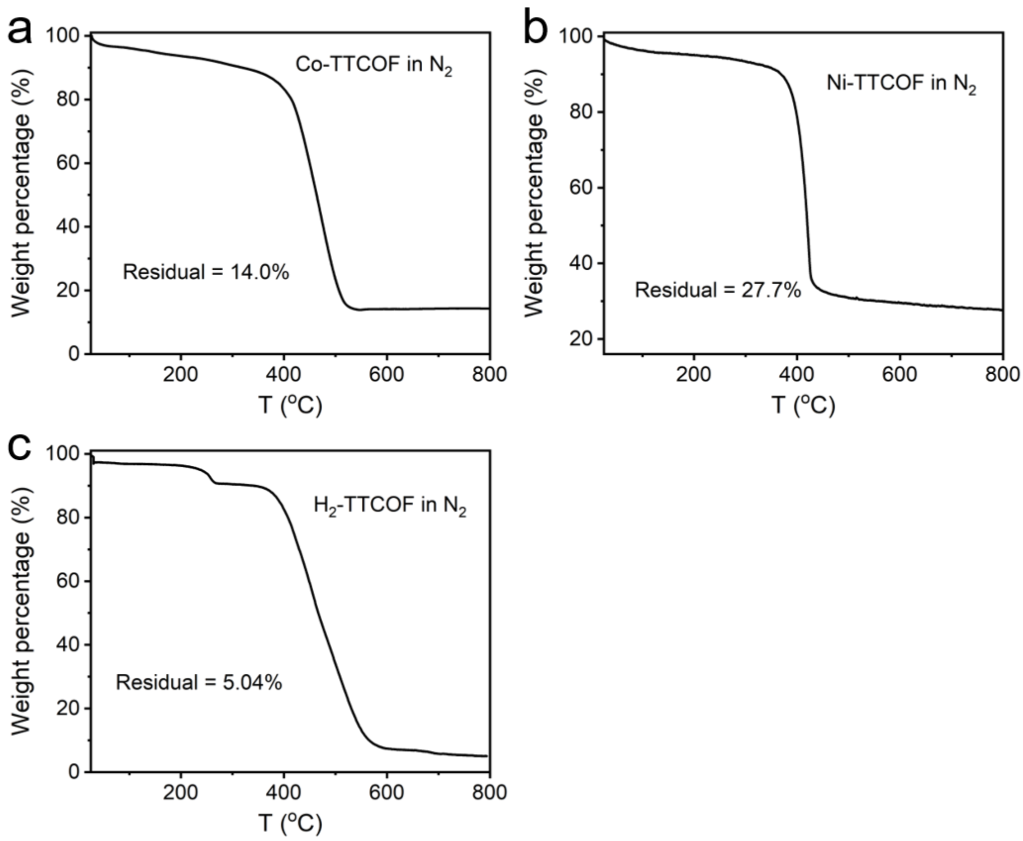

Supplementary Figure 18. TGA analyses of M-TTCOFs under N_2 atmosphere. **a** Co-TTCOF. **b** Ni-TTCOF. **c** H_2-TTCOF.

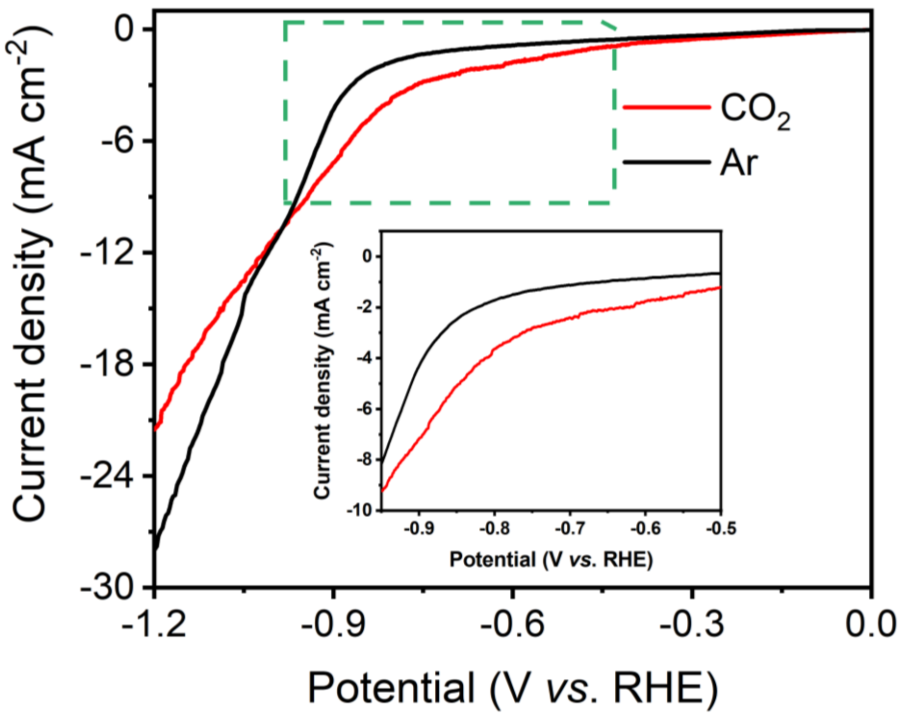

Supplementary Figure 19. Linear sweep voltammetric curves of Co-TTCOF tested in Ar-saturated and CO_2-saturated 0.5 M $KHCO_3$ aqueous solution.

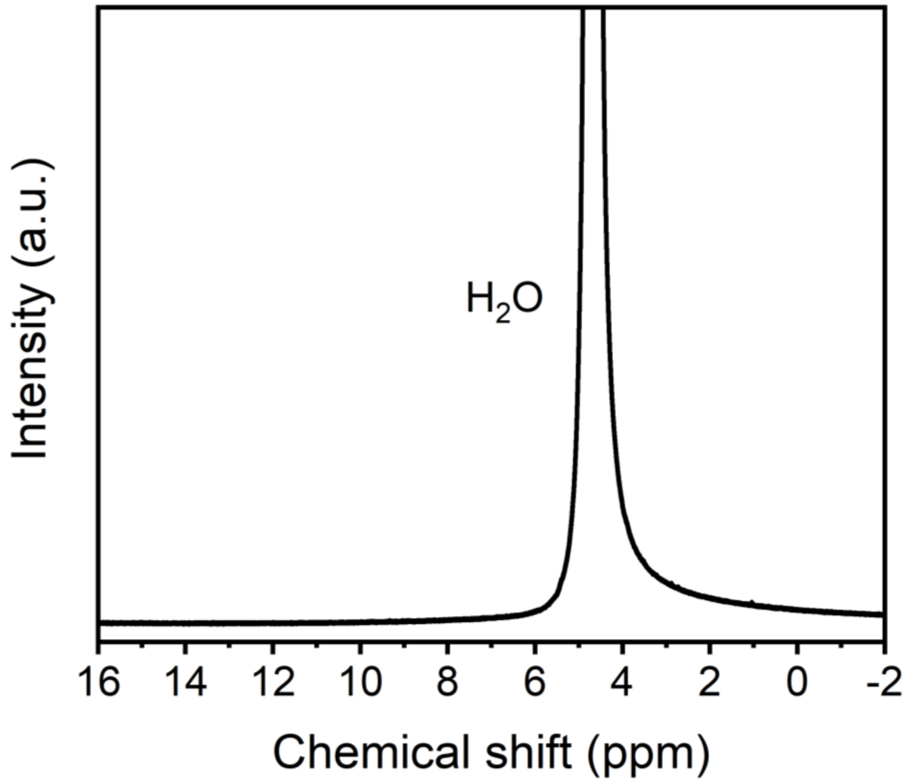

Supplementary Figure 20. ¹H NMR characterization of the liquid product during CO_2 reduction process (electrocatalyst, Co-TTCOF).

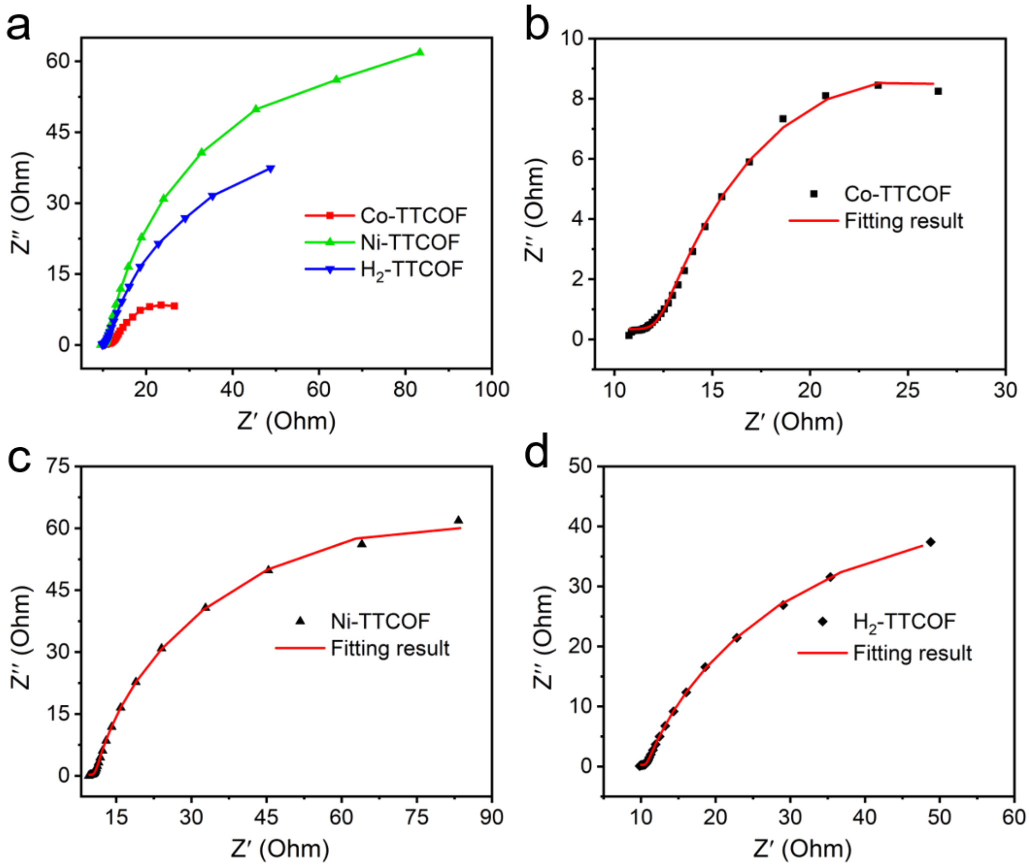

Supplementary Figure 21. a The Nyquist plots of M-TTCOFs and **b, c, d** fitting EIS spectrum of M-TTCOFs over the frequency ranging from 1000 kHz to 0.1 Hz at -0.7 V *vs*. RHE.

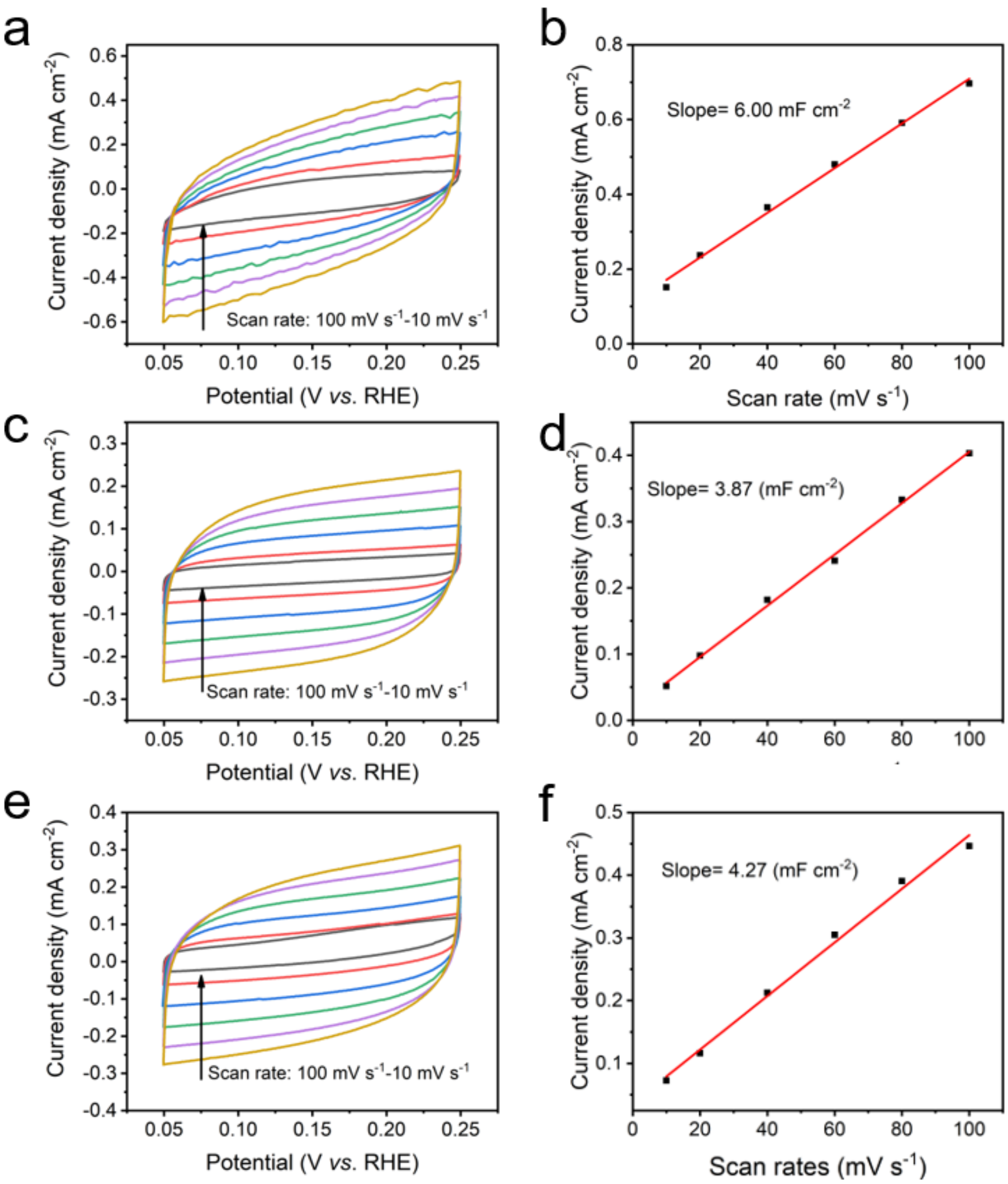

Supplementary Figure 22. Cyclic voltammogram (CV) curves in the region from 0.05 to 0.25 V vs. RHE at various scan rates (from 10 to 100 mV s^{-1}) and corresponding capacitive current at 0.15 V for **a, b** Co-TTCOF. **c, d** Ni-TTCOF. **e, f** H_2-TTCOF.

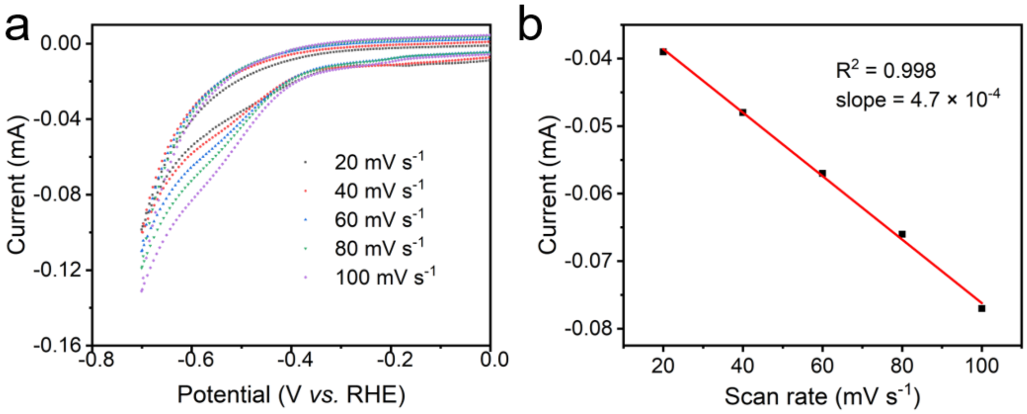

Supplementary Figure 23. The cyclic voltammogram tests of Co-TTCOF. **a** Cyclic voltammogram (CV) curves for Co-TTCOF. **b** Calculation of surface coverage (τ_0) for Co-TTCOF. Regression of the linear regime between 20 and 100 mV s^{-1} with equation: slope = n^2 F^2 A τ_0/4 R T (n = number of electrons involved; F = Faraday constant in C mol^{-1}; A = geometrical surface area of the electrode (0.071 cm^2); τ_0 = surface coverage; R = gas constant; T = temperature (298 K)).

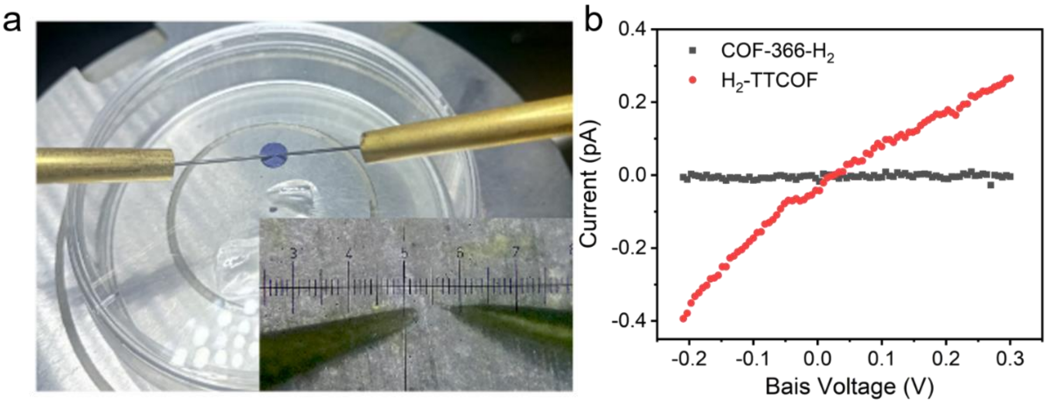

Supplementary Figure 24. *I*–*V* profile tests and instrument. **a** Test instrument. **b** *I*–*V* profile of H_2-TTCOF (red) and COF-366-H_2 (black).

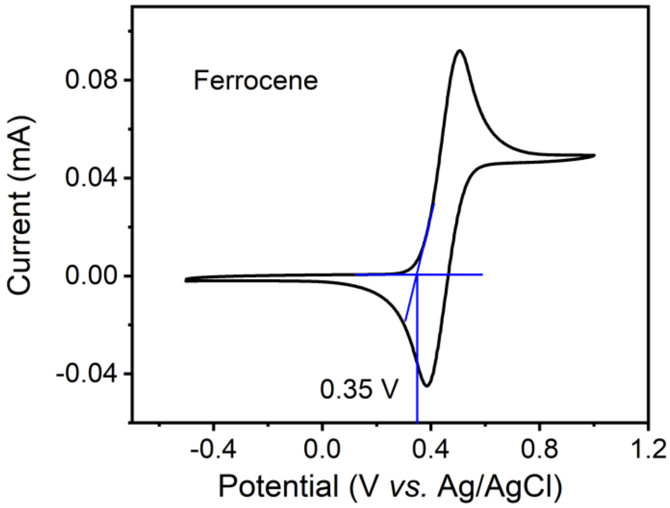

Supplementary Figure 25. Cyclic voltammograms of ferrocene (internal standard) in 0.1 M nBu_4NPF_6 in CH_3CN at room temperature (E(Fc/Fc^+) = 0.35 V).

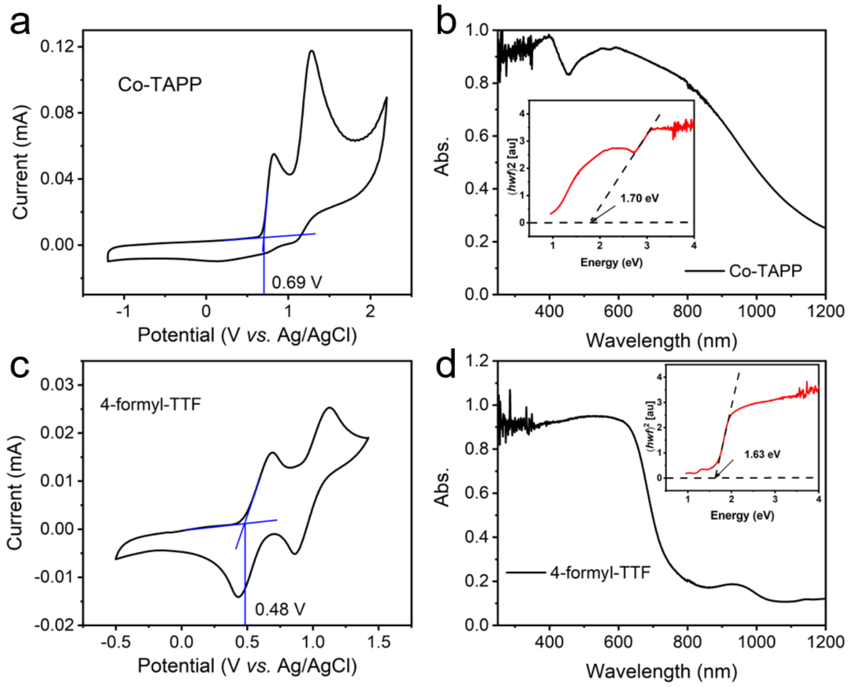

Supplementary Figure 26. The cyclic voltammogram and optical tests of Co-TAPP and 4-formyl-TTF. **a** Cyclic voltammograms of Co-TAPP. **b** Solid state UV of Co-TAPP (inset Tauc plot). **c** Cyclic voltammograms of 4-formyl-TTF. **d** Solid state UV of 4-formyl-TTF (inset Tauc plot).

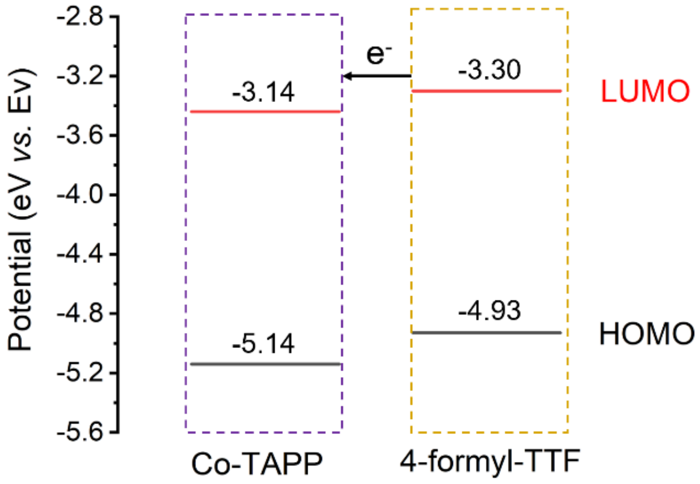

Supplementary Figure 27. LUMO (red) and HOMO (black) levels of Co-TAPP and 4-formyl-TTF.

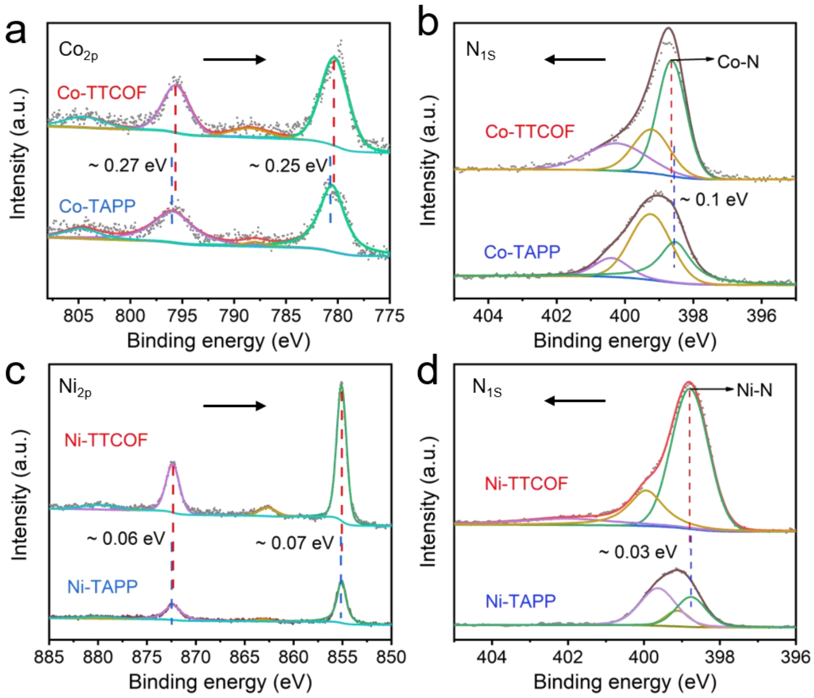

Supplementary Figure 28. High-resolution XPS spectrum of Co-TTCOF and Ni-TTCOF. **a** Co2p for Co-TTCOF. **b** N1s for Co-TTCOF. **c** Ni2p for Ni-TTCOF. **d** N1s for Ni-TTCOF. We have observed the shift in the binding energy of cobalt, nickel and N in COF structures compared with metallated porphyrin monomer. Co-TTCOF/Co-TAPP and Ni-TTCOF/Ni-TAPP have been selected as target pairs to distinguish the possible binding energy shift in XPS spectra. The spectra of Co-TTCOF and Co-TAPP display one pair of peaks arising from the spin-orbit doublet of Co2p, which can be assigned to the $Co2p_{3/2}$ and $Co2p_{1/2}$ (Supplementary Figs. 34a, b). The $Co2p_{3/2}$ and $Co2p_{1/2}$ peaks of Co-TTCOF locate at 780.84 eV and 796.14 eV, which presents apparent positive shift of ~0.25 eV and ~0.24 eV compared with pristine Co-TAPP ($Co2p_{3/2}$, 780.59 eV and $Co2p_{1/2}$, 795.90 eV). Besides, the binding energy of N1s for Co-TTCOF (Co-N bond, 398.64 eV) displays a negative shift of ~0.1 eV when compared with Co-TAPP (Co-N bond, 398.54 eV). Similar phenomenon is also detected for Ni-TTCOF, in which 0.07 and 0.06 eV positive shift are observed for $Ni2p_{3/2}$ and $Ni2p_{1/2}$ when compared with Ni-TAPP (Supplementary Figs. 34c, d). Also for N1s, a 0.03 eV shift is detected for Ni-TTCOF in contrast to Ni-TAPP. The change of binding energy provides direct evidence that the charge carrier migration pathway might be from TTF to M-TAPP (M = Co, Ni).

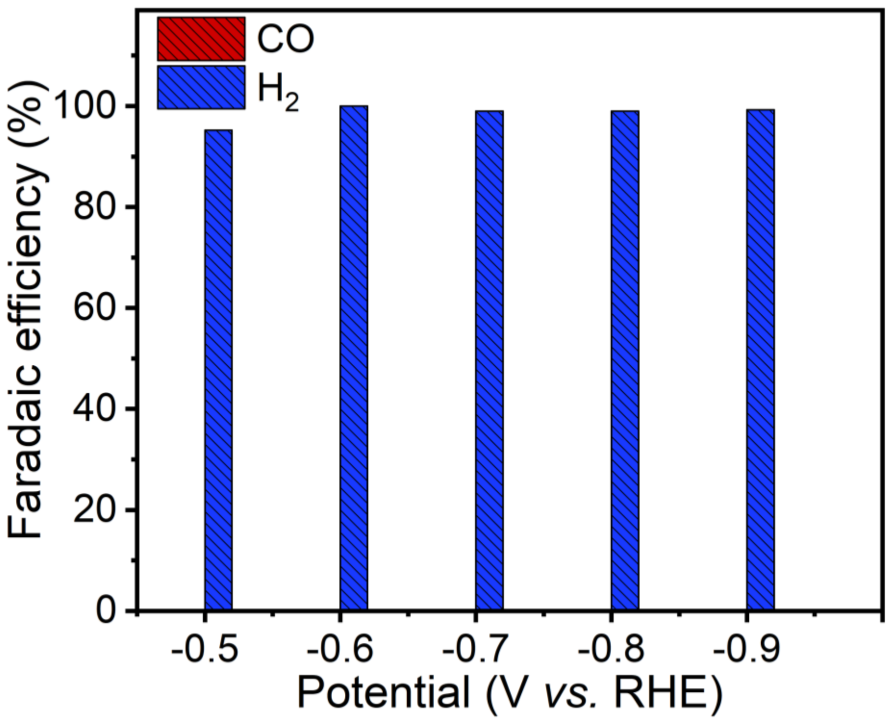

Supplementary Figure 29. FE_{CO} and FE_{H2} of Co-TTCOF at different applied potentials in Ar-saturated 0.5 M $KHCO_3$ aqueous solution.

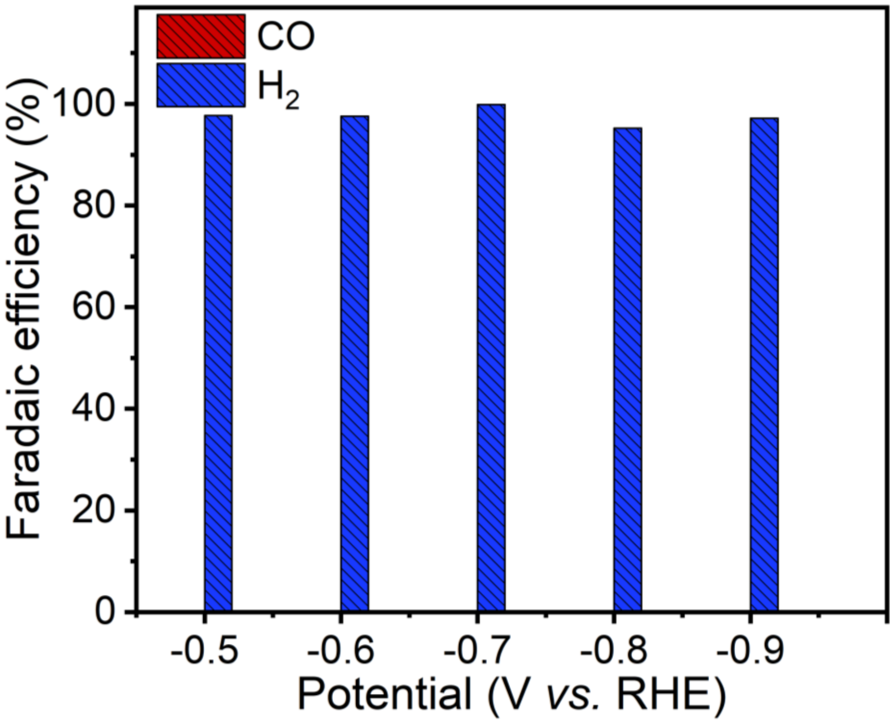

Supplementary Figure 30. FE_{CO} and FE_{H2} of pure carbon cloth at different applied potentials in CO_2-saturated 0.5 M $KHCO_3$ aqueous solution.

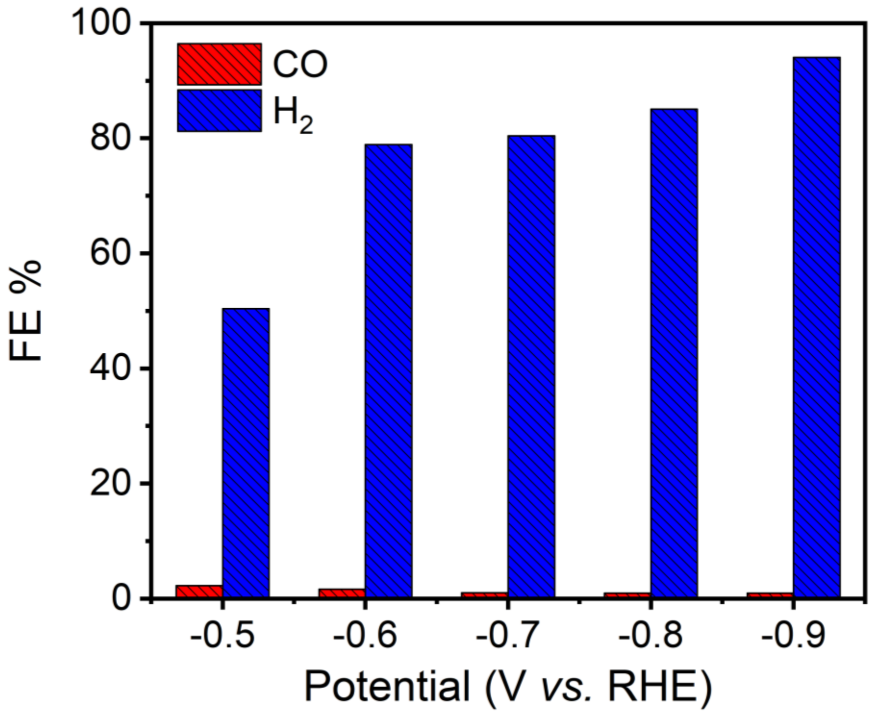

Supplementary Figure 31. FE_{CO} and FE_{H2} of the carbon cloth with acetylene black and Nafion at different applied potentials in CO_2-saturated 0.5 M $KHCO_3$ aqueous solution.

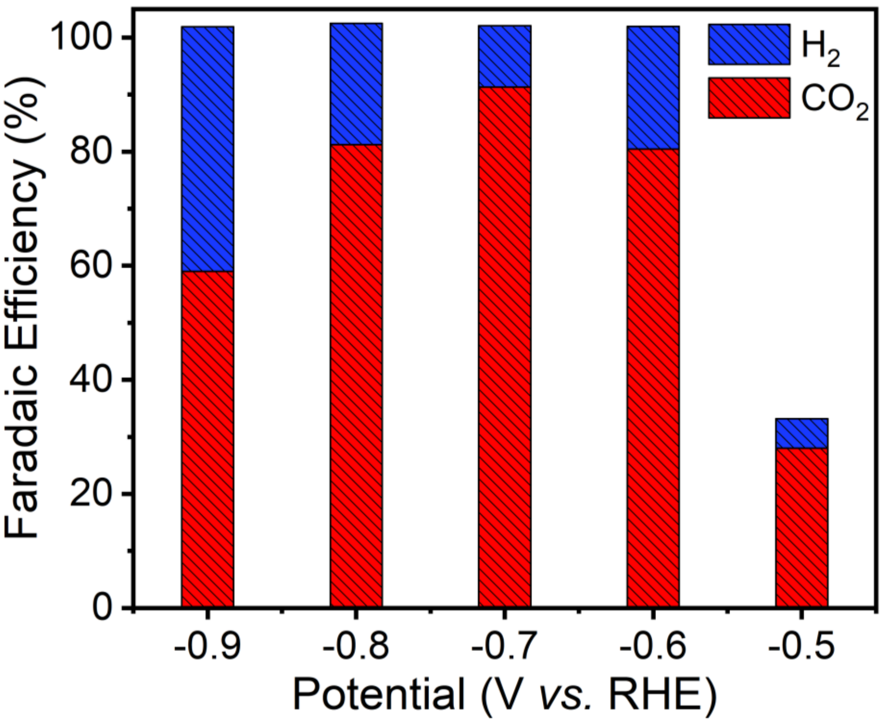

Supplementary Figure 32. FE_{CO} and FE_{H2} of Co-TTCOF at different applied potentials in CO_2-saturated 0.5 M $KHCO_3$ aqueous solution.

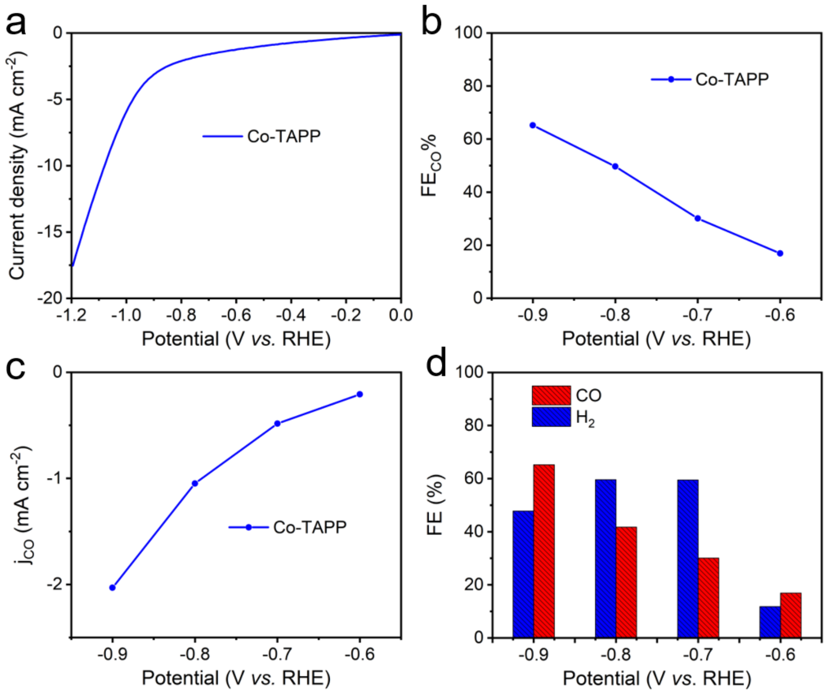

Supplementary Figure 33. Electrocatalytic performances of Co-TAPP. **a** LSV curves. **b** Faradaic efficiencies for CO. **c** Partial current density for CO. **d** FE_{CO} calculated over potential range from -0.5 to -0.9 V. Linear sweep voltammetry (LSV) curves (without *iR* compensation) show that the onset potential of Co-TTCOF (-0.45 V) is much more positive than that of Co-TAPP (-0.51 V) in CO_2-saturated $KHCO_3$ solution (Supplementary Fig. 33a). Furthermore, Co-TAPP exhibits a FE_{CO} of 69% at -0.9 V, which is inferior to Co-TTCOF (91.3%, -0.7 V) (Supplementary Figs. 33b, d). Besides, Co-TAPP gives a partial CO current density of 0.48 mA cm^{-2}, which is much less than that of Co-TTCOF (1.84 mA cm^{-2}) at -0.7 V (Supplementary Fig. 33c).

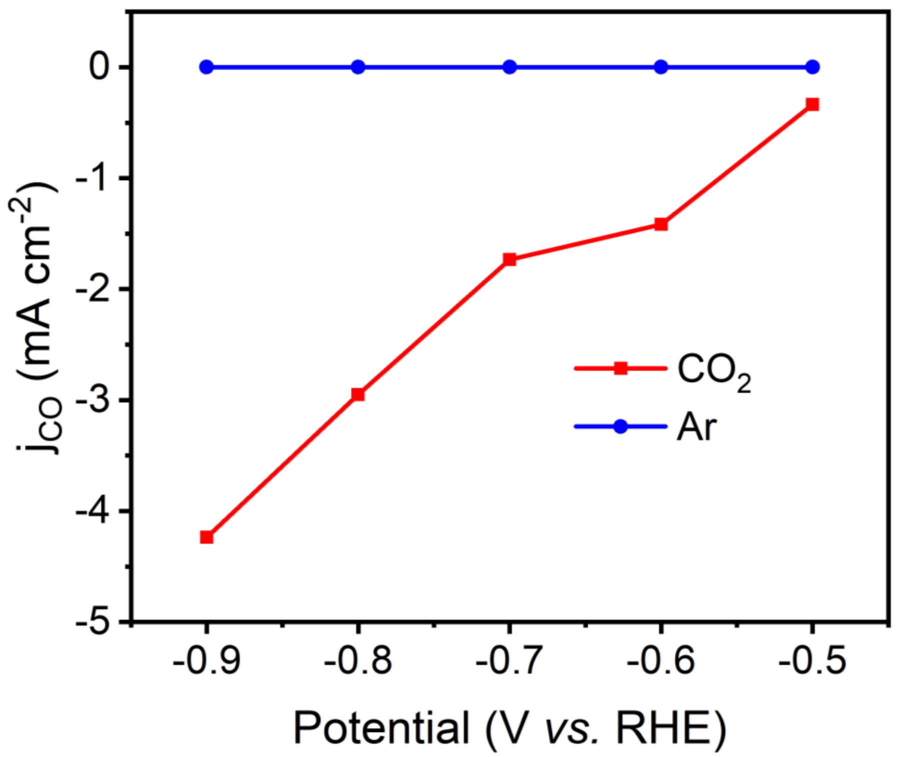

Supplementary Figure 34. CO partial current density of Co-TTCOF in Ar-saturated (blue) and CO_2-saturated (red) 0.5 M $KHCO_3$ aqueous solution.

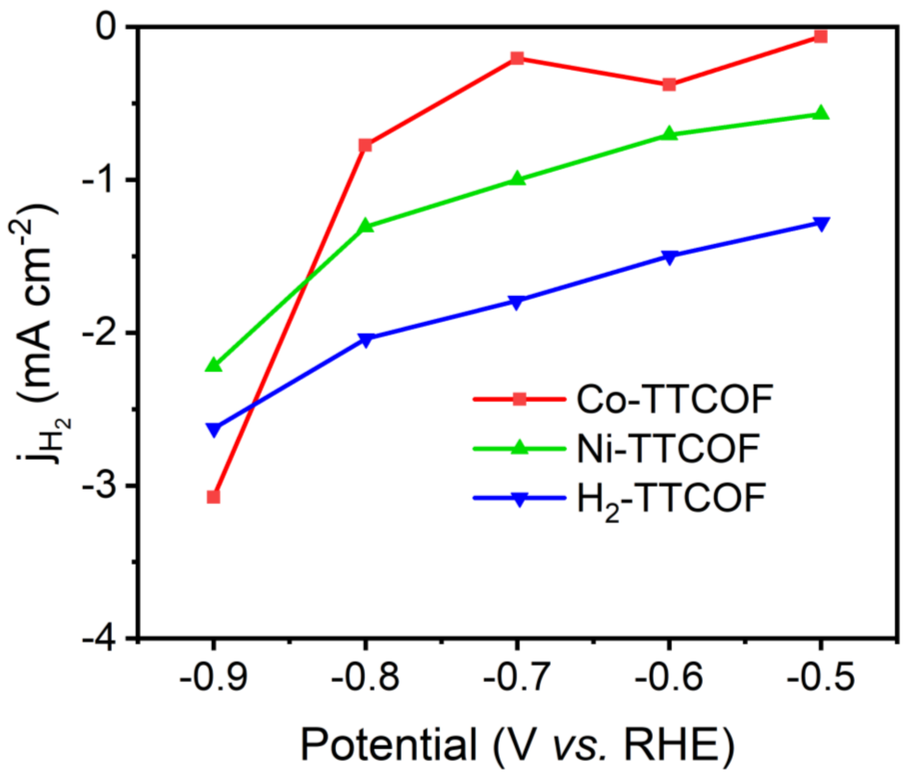

Supplementary Figure 35. Partial H_2 current density (based on geometric surface area) plots of M-TTCOFs in CO_2-saturated 0.5 M $KHCO_3$ aqueous solution.

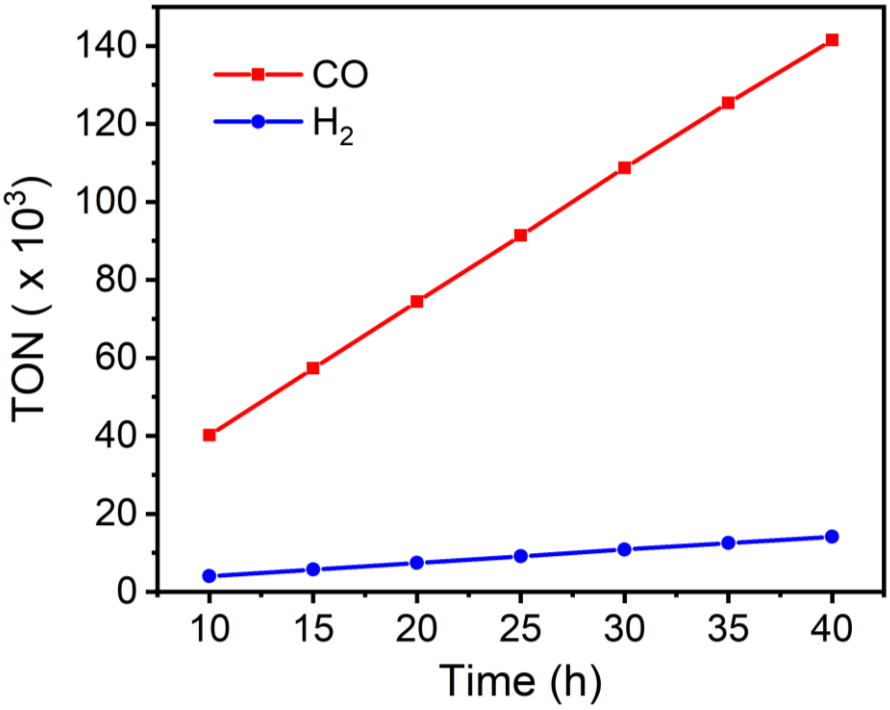

Supplementary Figure 36. Plots of CO and H_2 evolving turnover number versus time for Co-TTCOF. As shown in the images, the TON (CO) is as high as 40142 in just 10 h and can reach up to 141479 after 40 h.

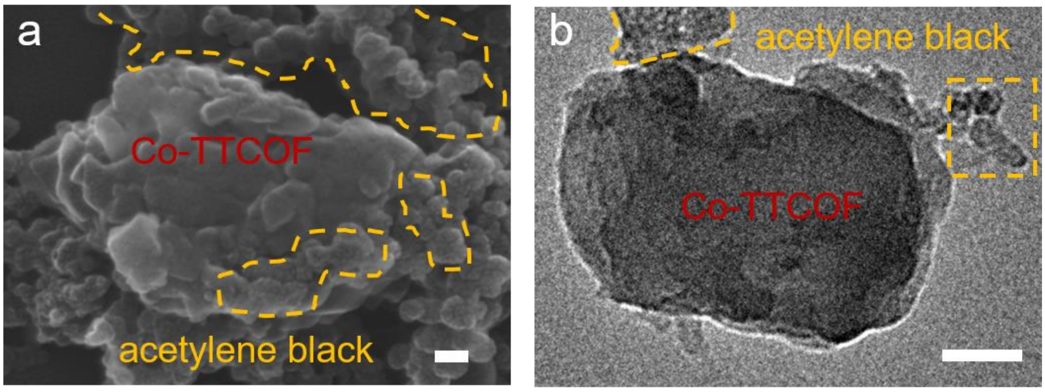

Supplementary Figure 37. SEM and TEM images of Co-TTCOF after long-time durability tests. **a** SEM image, scale bar = 100 nm. **b** TEM image, scale bar = 50 nm.

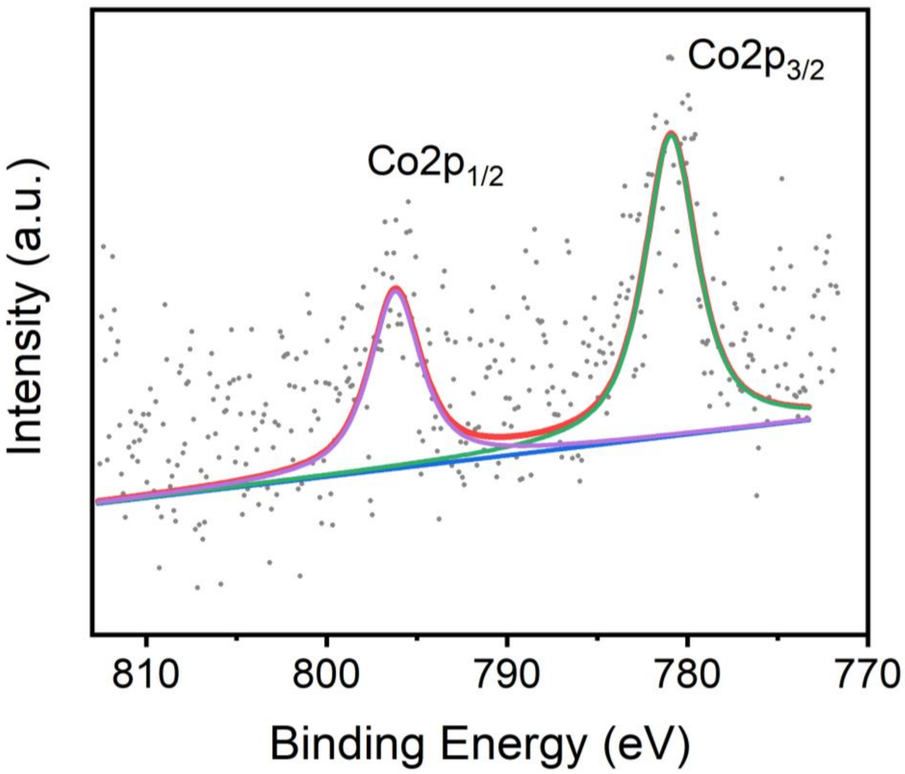

Supplementary Figure 38. High-resolution XPS spectrum of Co-TTCOF after long-time durability tests.

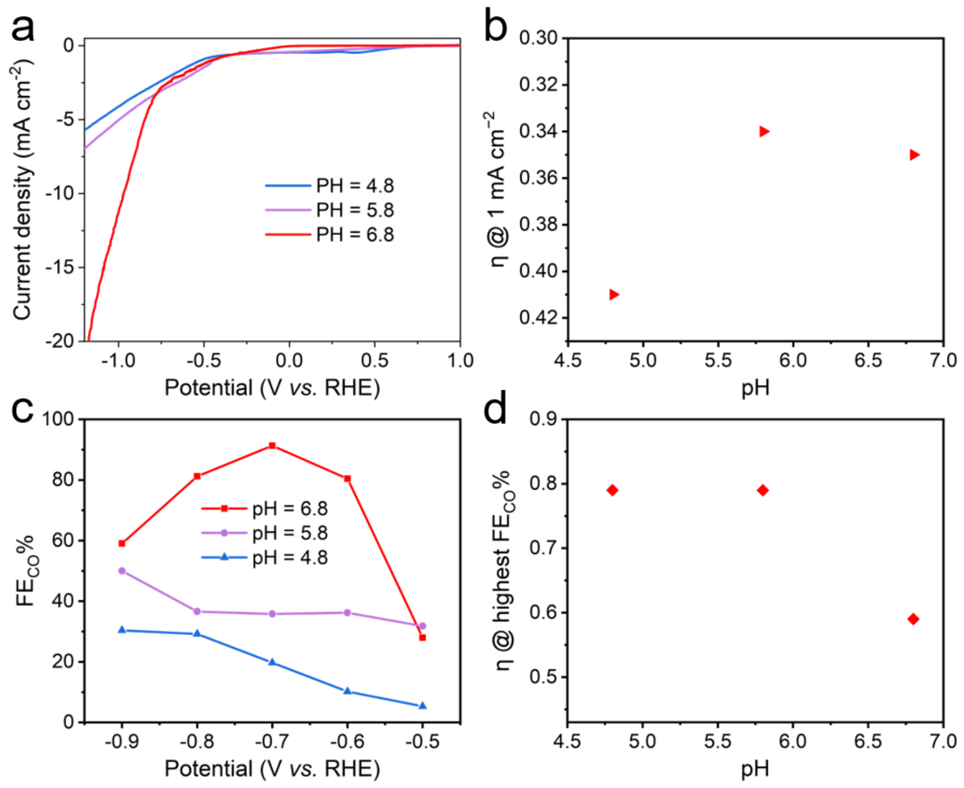

Supplementary Figure 39. The pH dependence of overpotential values for Co-TTCOF. **a** LSV curves. **b** Overpotential (η @ 1 mA cm^{-2}) vs pH plot. **c** FE_{CO} calculated over potential range from -0.5 to -0.9 V in various solution. **d** Overpotential (η @ highest FE_{CO}%) vs pH plot. Linear sweep voltammetry tests (LSV, without *iR* compensation) and electrocatalytic CO_2RR performances of Co-TTCOF as two kinds of powerful methods are applied to reveal the pH dependence of overpotential values from different aspects. In LSV curves, the overpotential (1 mA cm^{-2}) of Co-TTCOF decreases from ~410 mV (pH, 4.8) to ~340 mV (pH, 5.8) and finally slightly increases to ~350 mV (pH, 6.8) with the increase of pH values (Supplementary Fig. 39a,b). While for the electrocatalytic performances of Co-TTCOF, the overpotential (highest FE_{CO}%) of Co-TTCOF reaches to ~790 mV both for pH = 4.8 and pH = 5.8, then the value decreases to ~590 mV (pH, 6.8) (Supplementary Fig. 39c, d). The results of these two methods indicate that the overpotential value is closely related to the pH of electrolyte.

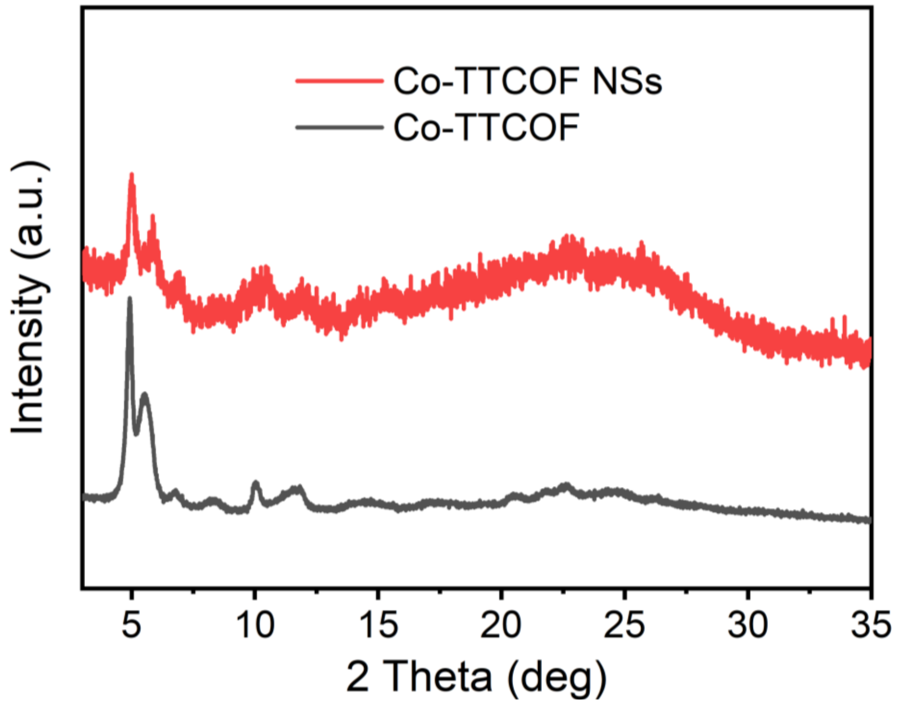

Supplementary Figure 40. The PXRD patterns of Co-TTCOF NSs.

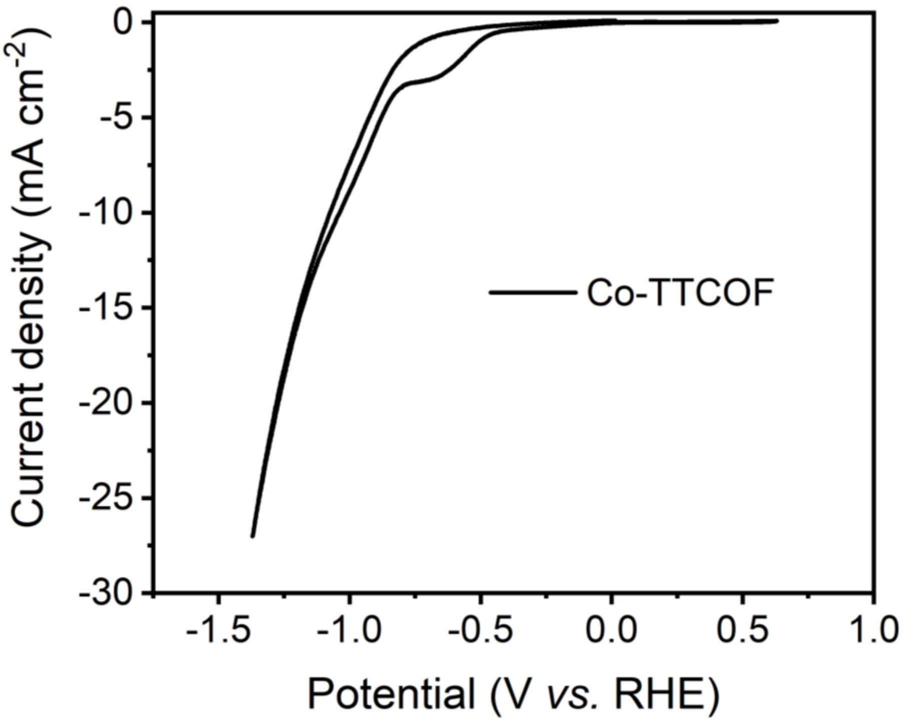

Supplementary Figure 41. CV curve of Co-TTCOF in N_2-saturated 0.5 M $KHCO_3$.

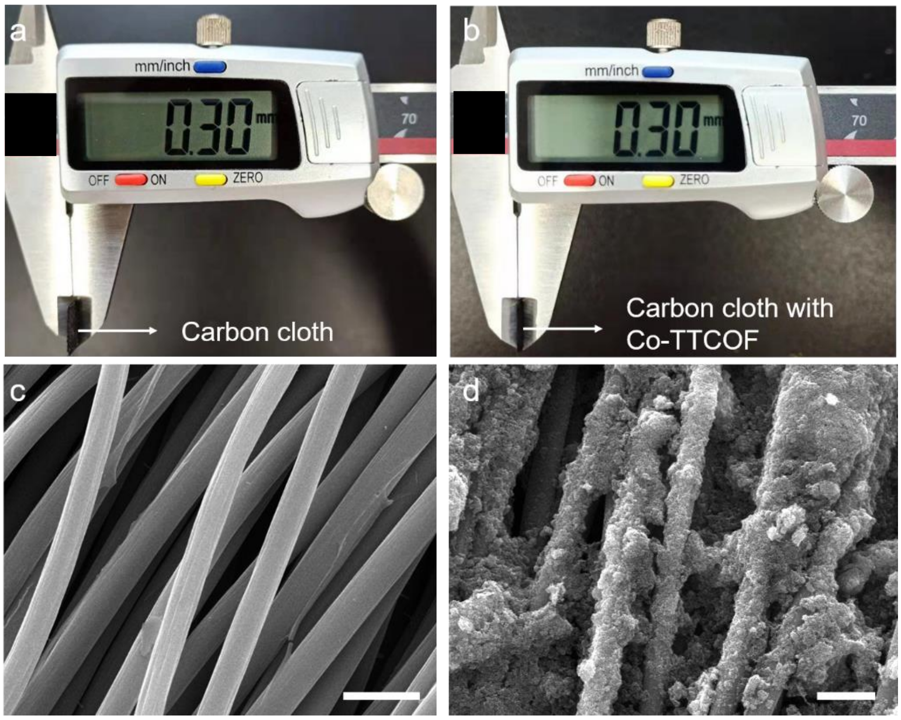

Supplementary Figure 42. The thickness test using a vernier caliper and SEM images. **a** The thickness test of carbon cloth using a vernier caliper (all the samples are properly seized on the vernier caliper without deformation). **b** The thickness test of working electrode using a vernier caliper. **c** The SEM image of carbon cloth, scale bar = 20 μm. **d** The SEM image of working electrode, scale bar = 20 μm.

Supplementary Table 1. Metal content analysis results of M-TTCOFs calculated from ICP tests.

Sample	Calcd. (wt%)	Found. (wt%)
Co-TTCOF	4.60%	3.37%
Ni-TTCOF	4.58%	3.17%

Supplementary Table 2. The summary of CO_2 electroreduction performances for reported electrocatalysts and M-TTCOFs.

Catalysts	Electrolyte	E (V *vs.* RHE)	Main product	FE (%)	Ref.
Co-TTCOF NSs	0.5 M $KHCO_3$	-0.8 V	CO	99.7	This work
Co-TTCOF	0.5 M $KHCO_3$	-0.7 V	CO	91.3	This work
Ni-TTCOF	0.5 M $KHCO_3$	-0.9 V	CO	20.9	This work
H_2-TTCOF	0.5 M $KHCO_3$	-0.7 V	CO	4.22	This work
COF-366-Co	0.5 M $KHCO_3$	-0.55 V	CO	90.0	1
COF-366-F-Co	0.5 M $KHCO_3$	-0.55 V	CO	87.0	2
COF-300-AR on Ag film	0.1 M $KHCO_3$	-0.85 V	CO	80.0	3
Cu nanosheets	2 M KOH	NA	acetate	48.0	4
single-atom iron	0.5 M $KHCO_3$	-0.47 V	CO	> 90	5
WSe_2 2D nanoflake	ionic liquid	-0.76 V	CO	> 80	6
Co_3O_4-CDots-C_3N_4	0.5 M $KHCO_3$	-0.6 V	CO	89	7
Cu-based nanoparticles	0.1 M $KHCO_3$	-1.1 V	C_2H_4	57.3	8
SnO_2 nanosheets	0.5 M $NaHCO_3$	-1.6 V (*vs.* Ag/AgCl)	$HCOO^-$	87	9

NA means not mentioned.

Supplementary Note 1: DFT calculations.

Computations were performed using the hybrid B3LYP DFT functionals. Basis sets of def2-TZVP[10,11] were adopted for all atoms in the complexes, and the DFT grid size was set to the standard m3 value. D3 dispersion correction developed by Grimme is included for weak interactions[12]. All of the DFT calculations were performed using the ORCA package employing the resolution of identity approximation[13].

Supplementary Note 2: The possible catalytic mechanism.

In N_2-saturated 0.5 M $KHCO_3$:

$Co(II)TTCOF + e \rightarrow Co(I)TTCOF^-$

$Co(I)TTCOF^- - e \rightarrow Co(II)TTCOF$

In CO_2-saturated 0.5 M $KHCO_3$:

$Co(II)TTCOF + e \rightarrow Co(I)TTCOF^-$

$Co(I)TTCOF^- + CO_2 \rightarrow *CO_2Co(II)TTCOF^-$

$*CO_2Co(II)TTCOF^- + H^+ + e \rightarrow *CO_2HCo(II)TTCOF^-$

$*CO_2HCo(II)TTCOF^- + H^+ \rightarrow Co(II)TTCOF + CO + H_2O$

Supplementary References

1. Lin, S., *et al.* Covalent organic frameworks comprising cobalt porphyrins for catalytic CO_2 reduction in water. *Science* **349**, 1208 (2015).
2. Wei, P.-F., *et al.* Benzoxazole-linked ultrastable covalent organic frameworks for photocatalysis. *J. Am. Chem. Soc.* **140**, 4623-4631 (2018).
3. Liu, H., Chu, J., Yin, Z., Cai, X., Zhuang, L., Deng, H. Covalent organic frameworks linked by amine bonding for concerted electrochemical reduction of CO_2. *Chem* **4**, 1696-1709 (2018).
4. Luc, W., *et al.* Two-dimensional copper nanosheets for electrochemical reduction of carbon monoxide to acetate. *Nat. Catal.* **2**, 423-430 (2019).
5. Gu, J., Hsu, C. -S., Bai, L., Chen, H. M., Hu, X. Atomically dispersed Fe^{3+} sites catalyze efficient CO_2 electroreduction to CO. *Science* **364**, 1091 (2019).
6. Asadi, M., *et al.* Nanostructured transition metal dichalcogenide electrocatalysts for CO_2 reduction in ionic liquid. *Science* **353**, 467 (2016).
7. Guo, S., *et al.* A Co_3O_4-CDots-C_3N_4 three component electrocatalyst design concept for efficient and tunable CO_2 reduction to syngas. *Nat. Commun.* **8**, 1828 (2017).
8. Jung, H., *et al.* Electrochemical fragmentation of Cu_2O nanoparticles enhancing selective C-C coupling from CO_2 reduction reaction. *J. Am. Chem. Soc.* **141**, 4624-4633 (2019).
9. Li, F., Chen, L., Knowles, G. P., MacFarlane, D. R., Zhang, J. Hierarchical mesoporous SnO_2 nanosheets on carbon cloth: a robust and flexible electrocatalyst for CO_2 reduction with high efficiency and selectivity. *Angew. Chem. Int. Ed.* **56**, 505-509 (2017).
10. Schäfer, A., Huber, C., Ahlrichs, R. Fully optimized contracted Gaussian basis sets of triple zeta valence quality for atoms Li to Kr. *J. Chem. Phys.* **100**, 5829 (1994).
11. Weigend, F., Häser, M., Patzelt, H., Ahlrichs, R. RI-MP2: optimized auxiliary basis sets and demonstration of efficiency. *Chem. Phys. Lett.* **294**, 143-152 (1998).
12. Grimme, S., Antony, J., Ehrlich, S., Krieg, H. A consistent and accurate ab initio parametrization of density functional dispersion correction (DFT-D) for the 94 elements H-Pu. *J. Chem. Phys.* **132**, 154104 (2010).
13. Neese, F. The ORCA program system. *Wiley Interdiscip. Rev.: Comput. Mol. Sci.* **2**, 73-78 (2012).
